# Adenia—with Report of Cases

**Published:** 1876-06

**Authors:** N. A. Drake

**Affiliations:** Ossian, Iowa


### ADENIA—WITH REPORT OF CASES.

By N. A. DRAKE, M.D., Ossian, Iowa.

We have but very little literature on the subject of this disease. Trousseau devotes a lecture of thirty-one pages to it. He it was who gave it the name—Adenia. It is a disease characterized by progressive hypertrophy of the lymphatic glands. Trousseau claims no priority in describing the disorder, but gives due credit to Hodgkin, of England, who described it as far back as 1832; and to-day it is known as Hodgkin's disease. The Royal College of Physicians, however, do not recognize it. Yet Aitken gives two pages in his work to its description, and regrets that he, among others, has aided in confounding it with lardaceous diseases.

Virchow describes the disease as lympho-sarcoma in his work on Tumors, which first appeared in 1865.

Wunderlich, I believe, adheres to the name of Hodgkin's disease.

Trousseau's lectures were not delivered on this subject until two years after Virchow's, and hence he was able to give the result of more study.

Little can be found, written this side of the Atlantic, on this disease.

Dr. W. H. Triplett read an essay on the subject before the Medical Society of the District of Columbia in 1874. In this he adheres to the name of Hodgkin's disease.

In July, 1869, I was called to see a Norwegian woman, about thirty years of age, who had lately arrived in this country. On first sight she presented a peculiar appearance. The cervical and submaxillary glands were so enlarged as to produce a very singular deformity, while she had a peculiar cachexia. Her breathing was labored, she had a dry, hacking cough, and complained of great constriction in her chest. The axillary glands were as greatly enlarged as those in the cervical and submaxillary regions. There was no tendency to suppuration in any of the tumors; they were not inflamed at all. I could manipulate them freely with no complaint from the patient. The glands in the inguinal region were not as much enlarged. There was considerable anasarca. On auscultation of the chest, I could detect nothing but exaggerated normal sounds.

I gleaned from her that she had first noticed these tumors about one year previous, and that they had been gradually enlarging. Her appetite was good, but she was considerably emaciated. The liver and spleen were not perceptibly enlarged.

She died the month following.

I now believe this to have been a typical case of adenia. I then contented myself with pronouncing it a case of scrofula. I possessed neither the book of Trousseau nor Aitken at the time.

In May, 1874, L. F., an American, something over thirty years of age, at that time a "runner" for a Chicago grocery house, consulted me for violent nocturnal pain in his abdomen. He said it had been troubling him occasionally for two or three months, and usually he obtained relief by hot fomentations. He looked careworn, as one does who has lost sleep and suffered pain. He was still traveling, and desired me to attend him so that he would not be obliged to suspend business. I gave him a casual examination, and thinking his trouble originated from more or less indigestion and irritability of bowels, I prescribed a laxative, to be followed by an anodyne; and as he was going to remain at home a few days, requested him to call and see me again before he went away. He did so, and reported himself considerably relieved. He went away on a trip but returned in a short time as bad as ever, and I then advised him to remain at home for awhile.

About this time he called my attention to some bunches in his neck, and said he had some under his arms and in his groins. Upon closer examination I found almost all of his superficial lymphatic glands slightly enlarged and none of them inflamed. This at once called to my mind the case just related.

Remembering that I had read something in Trousseau that seemed to describe his case, I turned to Adenia and was at once convinced that I had this disease with which to contend.

Having business that called him to Chicago, I advised him to consult Prof. Freer, not having given him yet an unfavorable prognosis.

He returned from Chicago in a week and told me that by the advice of a friend he had consulted Dr. Ludlam, a homœopathist. He said Dr. L. told him his difficulty was irritability of the bowels.

In about a month he returned and asked me to treat him. I then told him that his case was hopeless ; that his disease would eventually prove fatal. The disease was steadily advancing. The cervical and submaxillary glands were now noticeably enlarged, as were nearly all his superficial lymphatic glands; and I accounted for the nocturnal pain in his bowels, which continued, by the enlarged glands pressing on the nerve centers.

He was dissatisfied with my prognosis, and knowing of his confidence in Dr. Bulis of Decorah, I advised that he consult him.

Dr. Bulis confirmed my diagnosis, told him that medicine would be of no use, and advised him to go to some mineral spring.

He went back again to homœopathy, then to the most abject quackery.

For a time he looked better. He had now given up traveling, and considering himself improved, he opened a grocery store in our town.

The disease made steady progress, and I learned he was kept up on stimulants and tonics.

When cold weather came he grew rapidly worse, and then went to Hot Springs, Ark. ; returning, towards the spring of 1875, much worse.

On April 25th he sent for me to prescribe for him. He was very much emaciated ; had marked cachexia ; the pulse was somewhat accelerated ; respiration was difficult except when quiet ; all the lymphatic glands were very large ; there was extensive anasarca ; the urine was scanty ; the appetite very good. He was suffering no pain. I could do him no good.

He died May 16th, about one year from the time he first consulted me.

The glands in this case were not as large as in my first case. There was not much dropsy in the first case, while this was a very prominent symptom in the second, especially towards the last. The spleen was not enlarged in either case.

Trousseau reports eleven cases, and enlarged spleen occurred only in three, hence he infers that the spleen is not necessarily involved.

In both of my cases glandular enlargement was first noticed in the cervical region, and the affection appeared to travel successively downward.

In case of L. F. the first complaint was of nocturnal pain in the bowels. Neither Trousseau nor any other author I can find speaks of a parallel case. I think the pain was caused by the pressure of the enlarged glands on the nerves, especially when in the recumbent posture. I could distinctly feel these glands through the abdominal walls.

All cases of this disease, that I can find reported, have ended fatally. The duration of the disease is from a year to a year and a half.

